# Correlation Between Dental and Cervical Vertebral Maturation in Iranian Females

**DOI:** 10.5812/iranjradiol.9993

**Published:** 2012-12-27

**Authors:** Solmaz Valizadeh, Nakissa Eil, Sara Ehsani, Hooman Bakhshandeh

**Affiliations:** 1 Department of Oral and Maxillofacial Radiology, Faculty of Dentistry, Shahid Beheshti University of Medical Sciences, Tehran, Iran; 2 Department of Oral and Maxillofacial Radiology, Faculty of Dentistry, Tehran University of Medical Sciences, Tehran, Iran; 3 Dental Research Center, Research Institute of Dental Sciences, Shahid Beheshti University of Medical Sciences, Tehran, Iran; 4 Cardiovascular Intervention Research Center, Rajaie Cardiovascular Medical Research Center, Tehran University of Medical Sciences, Tehran, Iran

**Keywords:** Cervical Vertebrae, Radiography, Panoramic, Tooth Calcification

## Abstract

**Background:**

Considerable variations in the development stage among patients of the same chronological age have led to introduce the concept of the developmental age based on the maturation of different organs such as cervical vertebrae or teeth.

**Objectives:**

The purpose of this study was to investigate the correlation between the stages of tooth calcification and the cervical vertebral maturation in Iranian females.

**Patients and Methods:**

Four hundred females (age range, 8 to 14 years) participated in the study. To determine the dental maturational stage, calcification of the mandibular teeth except for third molars were rated according to the method suggested by Demirjian et al. To evaluate the stage of skeletal maturation, cervical vertebral morphologic changes were assessed on lateral cephalometric radiographs according to the method explained by Baccetti et al. Correlations between bone maturation and teeth calcification were showed by Spearman's correlation and Kendall’s tau-b coefficients. The relevant associations were investigated by ordinal logistic regression models.

**Results:**

Correlations between the two stages were observed in the first and second premolars, canine and central incisors. All these correlations were significant. The association between cervical vertebral maturation and tooth calcification was greatest in the lateral incisor (odds ratio (OR) = 11, 95% confidence interval (CI): 6.6-18.3). However, considering the 95% CI for OR, no significant difference was detected among the second molar, first molar and lateral incisor.

**Conclusion:**

The relationship between calcification of teeth and maturation of cervical bones was significant. Bone maturation can be predicted by using teeth calcification stages, especially in the second molar, first molar and lateral incisor.

## 1. Background

In orthodontic and dentofacial orthopedics, it is evident that the timing of the treatment onset may be as critical as the selection of a specific treatment plan. By starting the treatment at the patient’s optimal maturation stage, the best results with the least chance of treatment failure can be predicted (1). Since orthodontic interventions only modify the growth pattern and do not cause it, the best time for treatment depends on the most rapid growth period that can specifically help to modify the skeletal defects (2). Although chronological age is used to recognize the developmental stage, it is a weak growth predictor and physiological age would be more reliable to evaluate the maturation state (3).

Physiological age is estimated by various maturation indicators such as an increase in body height, skeletal maturation of the hand-wrist, dental development, menarche, voice changes and cervical vertebral maturation (4). At present, the use of hand-wrist radiographs is the most common way to evaluate skeletal maturation (5). However, because of the additional exposure needed, especially concerning the age of patients referred for orthodontic treatment, this indicator has evoked more concern in the recent years (1).

Another effective tool, which has been recently introduced to determine adolescent growth spurt, is the patient’s cervical vertebral maturation observed on lateral cephalometric radiographs (6-11). In 1972, Lamparski et al. suggested a method for evaluation of skeletal maturation considering morphological changes in the cervical vertebra (10). Hassel and Farman developed a six-stage- method for cervical vertebral maturation (11). Baccetti et al. have modified their method to five stages. The method introduced by Baccetti et al. has been proven reliable and valid enough to substitute the hand wrist analysis (8). Dental maturity is also an indicator of the biological maturity of growing children (12). Dental age can be based on dental eruption or on the stages of tooth calcification observed in radiographs. The latter is more reliable because it is based on distinct details of the tooth shape and it uses the ratio of root length to crown height. Furthermore, tooth eruption can be affected by some environmental factors (13, 14). The method introduced by Demirjian et al. is one of the common methods used to determine the stages of calcification in several teeth (15). This method uses an approach to classify tooth mineralization by maturation changes in tooth development rather than just enlargement of the tooth. Since the final tooth size can vary from individual to individual, this system provides a method to categorize teeth not on size, but on certain dental maturity stages that are recognizable (16).

Regarding assessment of dental development which is straightforward and time-saving, and the fact that periapical or panoramic radiographs are routinely used in most dental clinics, they potentially can be a versatile alternative for hand-wrist radiographs to assess individual maturity (17). The relationship between tooth calcification stages and skeletal maturity has been reported (18-20). Racial variations in these correlations have also been reported (19-21). There is no study about this correlation in Iranians.

## 2. Objectives

The objective of this study was to find the correlation between the developmental stages of mandibular teeth and cervical vertebral maturity stages in an Iranian female sample.

## 3. Patients and Methods

### 3.1. Patient Selection

A descriptive cross-sectional study was designed. The study protocol was approved in Iran Center for Dental Research (ICDR). Panoramic and lateral cephalometric radiographs of 400 female subjects, ranging in age from eight to 14 years, were scanned (MICROTEK Scan Maker, 9600XL, Taiwan R.O.C). The radiographs belonged to patients referred to the Department of Orthodontics, Faculty of Dentistry, Shahid Beheshti University of Medical Sciences. Sample selection was performed according to the convenient sampling method. The inclusion criteria were: (1) Iranian nationality; (2) no systemic disease that could affect general development like hormonal disease; (3) no history of orthodontic treatment; (4) lateral cephalometric and panoramic radiographs available with high clarity and good contrast taken in the same day; (5) no missing or anomalies (trauma, injury, impaction, transposition) in dentition (third molars were not included in this study); (6) no history of trauma or surgery in the neck or dentofacial region. We tried to select equal samples for different stages of the cervical vertebral maturation.

### 3.2. Imaging Studies

**Figure 1:**
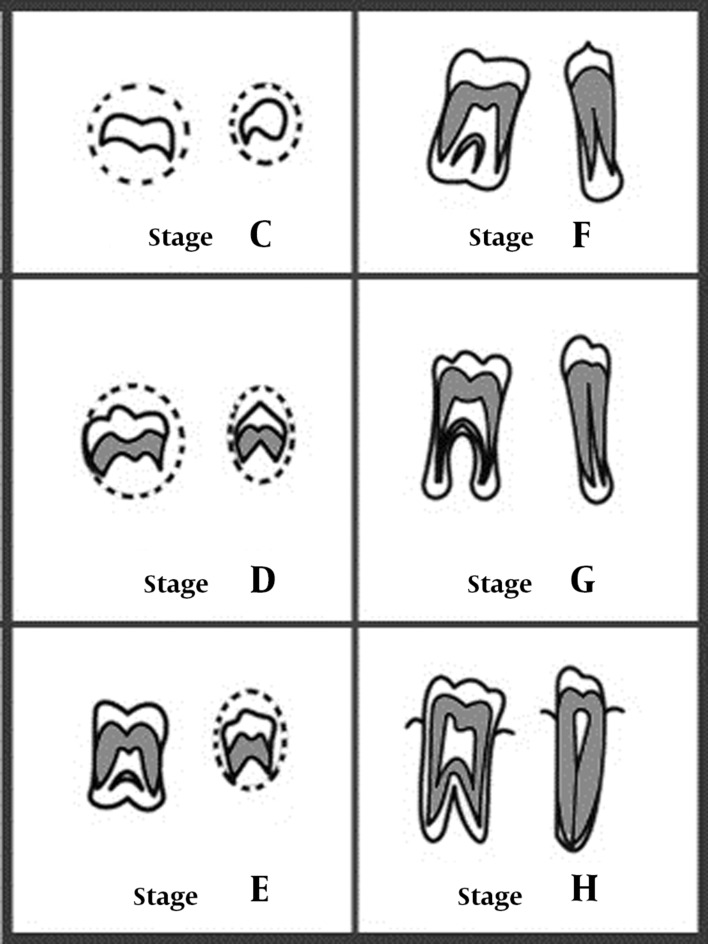
Demirjian tooth calcification stages C to H (15)

Panoramic radiographs were studied to determine the calcification stage of permanent mandibular teeth on the left side. We did not consider the maxillary teeth because superimposition of the anatomic structures in this area does not always allow assessment of the accurate developmental stage of the teeth. Third molars were not considered in the assessment. Tooth calcification stages were rated from A to H according to the method introduced by Demirjian et al. (Figure 1) (15). The characteristics of stages are described in Table 1.

**Table 1:** Stages of Tooth Calcification C to H, According Demirjian et al. (15)

	Description
**Stage C**	Complete enamel formation on occlusal surface, dentin formation started, curved pulp chamber, no pulp horns
**Stage D**	Complete crown formation to CEJ, root formation started, curved pulp chamber, pulp horns begin differentiation
**Stage E**	Root shorter than crown, straight pulp chamber walls, pulp horns more differentiated, radicular bifurcation calcification started
**Stage F**	Isosceles triangle form of the pulp chamber, the root length equal to or greater than crown, distinct root form because of sufficiently calcified bifurcation
**Stage G**	Parallel walls of the root canal, open apical end
**Stage H**	Completely closed root apex, uniform periodontal membrane surrounding the root and apex

Abbreviations: CEJ, cemento-enamel junction

**Figure 2:**
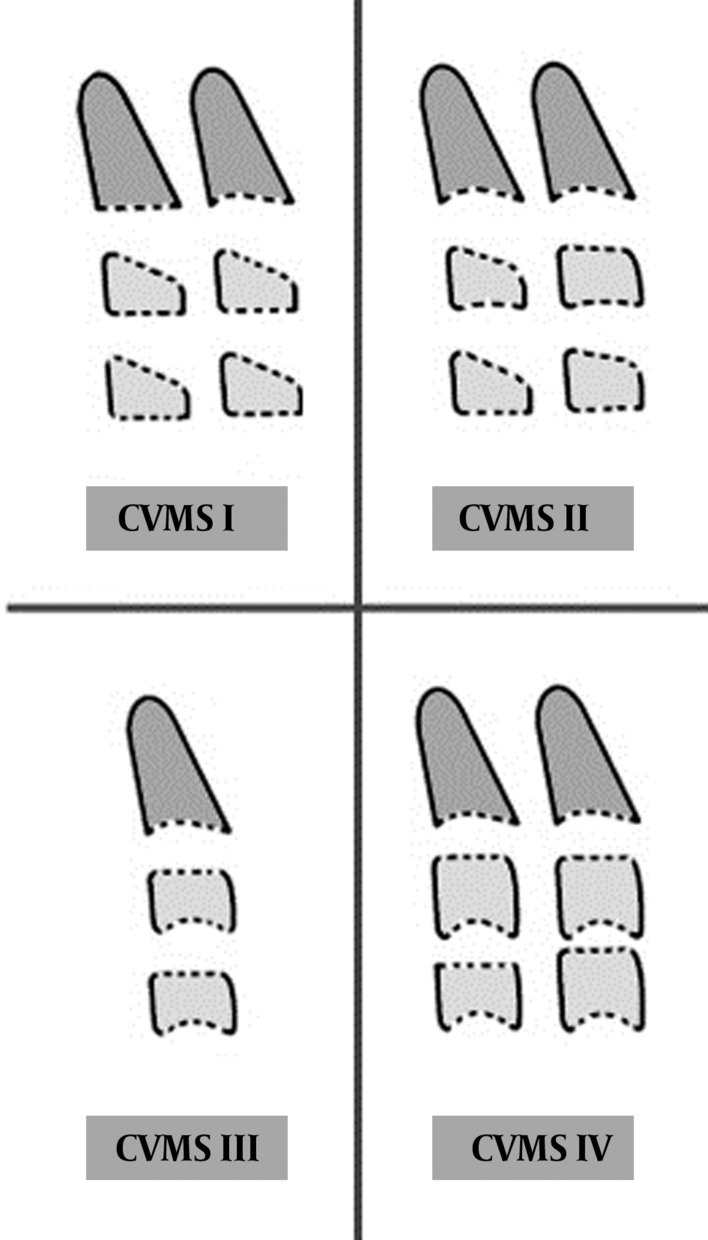
Cervical vertebral developmental stages, CVMS I to CVMS IV, proposed by Baccetti et al. (8).

Cervical vertebral maturation stage (CVMS) was evaluated on lateral cephalometric radiographs, according to the method described by Baccetti et al. (Figure 2, Table 2) (8). This method, known as CVMS, has been proved useful in the evaluation of skeletal maturation in a single cephalogram. This method analyzes the morphology of the second (C2), third (C3), and fourth (C4) cervical vertebrae and the patient is classified into one of five stages; CVMS I, CVMS II, CVMS III, CVMS IV and CVMS V which are demonstrated in Table 1. Since the radiographs were selected from existing data, there was no risk of additional radiation exposure to patients. All radiographs were analyzed by two maxillofacial radiologists who examined panoramic and lateral cephalometric radiographs separately. Cases with disagreement between the observers were excluded.

**Table 2:** Cervical Vertebral Development Stages, Proposed by Baccetti et al. (8)

	Shape of the Body (C3)	Shape of the Body (C4)	Lower Border of C2	Lower Border of C3	Lower Border of C4
**CVMS I**	Trapezoid	Trapezoid	Flat	Flat	Flat
**CVMS II**	Trapezoid or Rectangular horizontal	Trapezoid or Rectangular horizontal	Concave	Concave	Flat
**CVMS III**	Rectangular horizontal	Rectangular horizontal	Concave	Concave	Concave
**CVMS IV**	Rectangular horizontal or Squared	Rectangular horizontal or Squared	Concave	Concave	Concave
**CVMS V**	Rectangular vertical	Rectangular vertical	Concave	Concave	Concave

### 3.3. Intra-Observer Reliability

To examine the intra-observer reliability, 20 subjects were reevaluated by both methods. The agreement was assessed by weighted kappa statistics. Kappa was 0.89 ± 0.03 for determination of tooth calcification stages and 0.92 ± 0.05 for cervical maturation. The results revealed that the reproducibility of the diagnosis in our rater was almost perfect.

### 3.4. Statistical Analysis

Data were presented as percentage (%). The Spearman's correlation and Kendall’s tau-b coefficients were used to study the correlation between cervical vertebral maturation stages and dental calcification stages. The associations between the variables were assessed by Wilcoxon signed rank test. In addition, we used ordinal logistic regression models to define the associations between cervical and dental maturation stages. P values less than 0.05 were considered statistically significant. SPSS 15 for Windows (SPSS Inc., Chicago, IL, USA) and STATA 11 (STATA Inc., Texas, USA) were used for statistical analysis.

## 4. Results

### 4.1. Descriptive Statistics

The radiographs of 430 (age range, 8-14 years) participants were studied. Twenty-two of them were excluded because of not reaching a definite decision about the stage of cervical vertebral maturation. Only eight subjects were in stage V of Baccetti classification and because of inadequate subjects, this stage was not considered. The remaining 400 participants had a quite similar distribution among the first four stages. The number and percentage distribution of the subjects in each stage of cervical vertebral maturation from CVMS I to CVMS IV are shown in Table 3. Distribution of the calcification stages of individual teeth are shown in Table 4. Tables 5, 6, 7 and 8 show the percentage distribution of calcification of each tooth at the cervical maturation stages from CVMS I to CVMS IV. None of the following results include the incisors and the first molar. At CVMS I (Table 5), no tooth calcification stage in none of the teeth had a percentage distribution greater than 50%. The first and second premolars in stage E showed the highest percent distribution (45.4% and 46.3%, respectively).

**Table 3:** Distribution of Cervical Vertebral Maturation in the Study Population (n = 400)

Stage	No. (%)
**CVMS I**	108 (27)
**CVMS II**	75 (18.75)
**CVMS III**	105 (26.25)
**CVMS IV**	112 (28)

**Table 4:** Descriptive Statistics of Calcification Stages for Each Studied Tooth (n = 400)

Stage	Central Incisor, No. (%)	Lateral Incisor, No. (%)	Canine, No. (%)	First Premolar, No. (%)	Second Premolar, No. (%)	First Molar, No. (%)	Second Molar, No. (%)
**C**	0 (0)	0 (0)	0 (0)	0 (0)	1 (0.3)	0 (0)	1 (0.3)
**D**	0 (0)	0 (0)	3 (0.8)	11 (2.8)	29 (7.3)	0 (0)	60 (15)
**E**	0 (0)	1 (0.3)	53 (13.3)	73 (18.3)	89 (22.3)	0 (0)	73 (18.3)
**F**	3 (0.8)	2 (0.5)	57 (14.3)	78 (19.5)	67 (16.8)	2 (0.5)	67 (16.8)
**G**	32 (8)	42 (10.5)	82 (20.5)	80 (20)	121 (30.3)	72 (18)	159 (39.8)
**H**	365 (91.3)	355 (88.8)	205 (51.3)	158 (39.5)	93 (23.3)	326 (81.5)	40 (10)

**Table 5:** Percentage Distribution of Calcification Stages of Teeth at CVMS I

Stage	Central Incisor, %	Lateral Incisor, %	Canine, %	First Premolar, %	Second Premolar, %	First Molar, %	Second Molar, %
**C**	0	0	0	0	0.9	0	0.9
**D**	0	0	2.8	10.2	19.4	0	39.8
**E**	0	0.9	35.2	45.4	46.3	0	32.4
**F**	2.8	1.9	32.4	27.8	21.3	1.9	17.6
**G**	22.2	27.8	17.6	11.1	9.3	44.4	9.3
**H**	75	69.4	12	5.6	2.8	53.7	0

**Table 6:** Percentage Distribution of Calcification Stages of Teeth at CVMS II

Stage	Central Incisor, %	Lateral Incisor, %	Canine, %	First Premolar, %	Second Premolar, %	First Molar, %	Second Molar, %
**C**	0	0	0	0	0	0	0
**D**	0	0	0	0	10.7	0	17.3
**E**	0	0	18.7	29.3	40	0	33.3
**F**	0	0	20	33.3	24	0	22.7
**G**	9.3	12	36	30.7	24	25.3	24
**H**	90.7	88	25.3	6.7	1.3	74.7	2.7

**Table 7:** Percentage Distribution of Calcification Stages of Teeth at CVMS III

Stage	Central Incisor, %	Lateral Incisor, %	Canine, %	First Premolar, %	Second Premolar, %	First Molar, %	Second Molar, %
**C**	0	0	0	0	0	0	0
**D**	0	0	0	0	0	0	3.8
**E**	0	0	1	1.9	8.6	0	10.5
**F**	0	0	6.7	21	20	0	20
**G**	1	2.9	27.6	28.6	49.5	4.8	61.9
**H**	99	97.1	64.8	48.6	21.9	95.2	3.8

**Table 8:** Percentage Distribution of Calcification Stages of Teeth at CVMS IV

Stage	Central Incisor, %	Lateral Incisor, %	Canine, %	First Premolar, %	Second Premolar, %	First Molar, %	Second Molar, %
**C**	0	0	0	0	0	0	0
**D**	0	0	0	0	0	0	0
**E**	0	0	0	0	0	0	1.8
**F**	0	0	0	9	4.5	0	8.9
**G**	0	0	6.3	13.4	36.6	0	58.9
**H**	100	100	93.8	85.7	58.9	100	30.4

At CVMS II (Table 6), a wide distribution of tooth calcification stages can be clearly seen in all of the teeth, with less than 50% in each stage. Stage E of the second premolar showed the highest percent distribution (40%). At CVMS III (Table 7), stage H of the canine (64.8%) and stage G of the second molar (61.9%) showed the highest percentage distribution. Tooth calcification stages in other teeth had a percentage distribution less than 50%. At CVMS IV (Table 8), stage H of canine (93.8%) and stage H of the first premolar (85.7%) had the highest percentage distribution.

### 4.2. Relationship Between Cervical Vertebral Maturation and Tooth Calcification Stages

Both the association and linear correlation between cervical vertebral maturation and the stages of tooth calcification are presented in Table 9. Greater correlations between the two stages were observed in the first and second premolar, canine and central incisor (Table 9).

**Table 9:** Correlation and Associations Between Calcification in Different Teeth and Cervical Maturation Stages

	Spearman’s rho a	Kendall’s tau-b a	Ordinal Logistic Regression Model b		
			Odds Ratio	95% CI for Odds Ratio	P Value
**Central Incisor**	0.71	0.61	3.9	3.2-4.8	< 0.001
**Lateral Incisor**	0.47	0.43	11.0	6.6-18.3	< 0.001
**Canine**	0.73	0.63	4.3	3.5-5.2	< 0.001
**First Premolar**	0.75	0.66	5.0	4.0-6.2	< 0.001
**Second Premolar**	0.71	0.62	4.7	3.7-5.9	<0.001
**First Molar**	0.37	0.33	9.9	5.2-18.9	< 0.001
**Second Molar**	0.34	0.31	10.7	5.1-22.3	< 0.001

Abbreviations: CI, confidence interval

^a^All P values < 0.001

^b^Calcification stage of teeth was the predictor and cervical maturation stage was the dependent variable.

According to the results of ordinal regression models, point estimation for odds ratio (OR) of the association between cervical vertebral maturation and tooth calcification was greatest in the lateral incisor (OR = 11, 95% CI: 6.6-18.3). However, considering the 95% CI for OR, no significant difference was seen between the second molar, first molar and lateral incisor (Table 9). It can be concluded that calcification stages in these teeth are the best predictor for the bone maturation state.

## 5. Discussion

The orthodontist should consider both the diagnostic records and the growth potential of the jaws. Although orthodontic treatment is able to modify the jaw growth and improve the dentofacial relationships, its ability is limited to the extent of jaw growth that might occur. Many investigators have studied the optimal time for treating patients with orthodontic functional appliances and it is known that periods of accelerated growth can contribute to correct those kinds of skeletal imbalances (2, 22). The pubertal spurt in growth can be assessed by some indicators such as increase in body height, (23, 24) skeletal maturation of the hand-wrist (5) and cervical vertebral maturation (6-11). In this study, we investigated the correlation between cervical vertebral maturation stages and the calcification stages of various teeth in Iranian females to know if there is a correlation between the tooth calcification stages and the CVM method. If this is true, a single panoramic radiograph which is of routine use may be suggested as an alternative to other methods that require further radiation exposure to the patients.

In this study, panoramic radiographs were used to evaluate dental maturity because they are usually available in orthodontic clinics and the mandibular region is clearly visible (17). The method introduced by Demirjian et al. was the method chosen to determine the tooth calcification stage (15). They classified tooth mineralization with regard to maturational changes rather than just increase in the length of the tooth, because of the wide variety of tooth sizes and also the radiographic magnification. This method consists of distinct details based on tooth shape and the ratio of root length to crown height rather than on the absolute length, so that foreshortened or elongated projections of developing teeth will not influence the reliability of assessment (19). This method uses tooth calcification rather than tooth eruption. The first disadvantage of eruption-based methods is that its exact timing cannot be determined (14-25). Moreover, it can be affected by local factors, systemic diseases and nutritional habits; therefore, their reliability may be questionable (15).

To evaluate the skeletal maturity, morphology of the second, third and fourth cervical vertebrae were assessed according to the method proposed by Baccetti et al. (8). The mandibular growth peak occurs between CVMS II and CVMS III, and it will not be reached without the accomplishment of CVMS I and CVMS II. This method has a comparable high reliability and validity as the hand-wrist analysis and it has no additional exposure to the patient (8). These three vertebrae are usually visible even when a protective radiation collar is worn. It anticipates the occurrence of mandibular growth peak which happens between CVMS II and III. Finally, every stage can be identified on a single cephalogram (1).

Only female subjects were studied in this investigation, because an important factor that affects the timing of adolescent growth spurt is the individual’s gender (3, 26-28). The velocity of the parapubertal growth spurt in girls is less than boys and happens at an average of two years earlier in life (26). This age difference in the beginning of the parapubertal growth spurt adds to the sexual diversity in physiological maturity (16). By choosing only female subjects, we tried to increase the internal validity of this study. Hägg and Taranger found that the onset of the pubertal growth spurt in height occurs around 10 years of aging girls (29). Björk and Helm also found that maximum pubertal growth in stature occurred in girls at the average age of 12.6 (30). So the age range of 10 to 12 was considered as the maximum growth spurt age and 8-14 years was the age range in this study.

The Spearman rank order correlation coefficients between the dental calcification and cervical vertebral maturation stages – except for the permanent incisors and first molar – were found to be high (0.702-0.75) which means a significant correlation between the two classifications exists (P < 001). Because apex closure occurs in the permanent incisors and the first molar, earlier than the age criteria of this study, the correlation coefficients for these teeth were 0.3 and 0.4, respectively. The first premolar showed the highest correlation. Krailassiri et al. reported the highest correlation in Thai individuals in the second premolar (19) and in Turkish subjects; Uysal et al. reported the highest correlation in the second molar (20). Some researchers suggested that the racial variation, nutrition, socioeconomic levels, and urbanization are causative factors of the differences in this correlation.(31, 32). Since this correlation has not been investigated in Iranian samples, this study was performed to assess the correlation in this group. Additionally, using different methods to evaluate skeletal maturity essentially results in different outcomes. Third molars were not considered part of the dentition because of the low correlation reported in previous studies (19, 20).

Previous studies have shown that a close relationship exists between tooth mineralization stage G and the appearance of the sesamoid bone. Therefore, they suggested this method for assessing the onset of puberty growth spurt via periapical or panoramic radiographs (22, 33). Krailassiri et al. (19) found a low relationship between early ossification of the sesamoid bone (which shows adolescent growth spurt) and dental calcification stage G. Our findings also showed a low relationship between adolescent growth spurt and dental calcification stage G.

Uysal et al. reported that root formation of the canine and the first premolar was completed in most cases (71%-80%) in pubertal growth spurt. In this study, in most of the subjects (64.8%), root formation of the canine was completed in Baccetti's CVMS III, in which the peak of mandibular growth is supposed to happen within the last one or two years. In 2011, Kalinowska et al. reported a moderate, but significant correlation between Demirjian’s dental stages and CVM in girls. The teeth showing the highest relationship with CVM was the second premolar. The central incisor had the least correlation. They suggested the use of dental calcification stages as a simple first-level diagnostic test to assess the skeletal maturity of patients (32).

The results of the present study suggested that the relationship between calcification of teeth and maturation of cervical bones are significant and bone maturation can be predicted by using the information of teeth calcification stages. The more useful teeth for this purpose are the second molar, first molar and lateral incisor.
